# The Princess and the Second Thousand

**Published:** 1891-08-01

**Authors:** 


### The Hospital

A Weekly Journal of

Science, Medicine, Nursing, and Philanthropy.

*For Hospitals, Asylums, Infirmaries, Dispensaries, Medical Practitioners, Students, Nurses, and the Charitable Public.*

Vol. X.—No. 253.] [Aug. 1, 1891.

### The Princess and the Second Thousand.

The most sanguine friend of the Royal National Pension Fund for Nurses never anticipated that, after the reception of the first thousand nurses by Her Royal Highness the Princess of Wales at Marlborough House in 1890, a second thousand would be ready to claim a similar honour in 1891. But so it has proved. The Pension Fund in itself, and apart from its benevolent endowments, is a much needed and excellent institution. With its £50,000 endowment, and its prospects of large increase in this direction, it assumes the character and proportions of a Nurses' Providence. The Princess of Wales has, from the first, clearly discerned the value of the Fund, and has taken the most effective steps to impress her judgment upon the nursing profession. The result of Her Royal Highness's firm and decided action has been that the Pension Fund has progressed with a rapidity and energy unparalleled in the annals of similar institutions.

Saturday, July 25th, was for nurses a day of days. The English climate, truly feminine in its versatility, seems resolved to be constant to the Princess of Wales and her protégées, the nurses. The day when the first thousand were presented dawned cloudy and dull, but during the hours of the ceremony the sun shone brightly, and all went merry as a marriage bell. The week in which the second thousand were to appear was showery and uncertain; but the presentation day itself was perfect in the balminess of its atmosphere and the radiance of its July sunshine. It was entirely a nurses' day: caps, aprons, starched strings, and uniforms were everywhere in evidence. The few visitors who were present by invitation merely served to bring into greater prominence the marked features of the occasion. The lawn and gardens of Marlborough House became for the time a nurses' promenade.

The nurses of Great Britain and the colonies owe a real debt of gratitude to the Prince and Princess of Wales for showing them where their true interests lie. All people who live by their own exertions have two kinds of provision to make: one for the present, the other for the future. When the Pension Fund was first started on its journey it was quite a new movement; and, like all new movements, it excited a good deal of criticism, some of which was markedly hostile. The nurses, having but small savings, were full of anxious fears about the investment of them. When they saw rival newspapers, and heard rival orators, contradicting each other with the most positive assurance, they did not know which way to turn for safe guidance. Their Royal Highnesses the Prince and Princess of Wales gave them a lead in the right direction at a most critical moment. The nurses felt that they could not be wrong if they adventured their fortunes with a vessel of which the Princess of Wales was, if not captain, at least Lord High Admiral, so to speak. Their faith in the Royal leadership was so great, that before the expiration of thirteen monthsfrom the time of the presentation of the first thousand, more than another thousand were found ready to take their places in the ranks at Marlborough House.

The Prince of Wales, in his speech, assured the nurses that the "Princess takes the deepest personal interest in this most beneficent society." The interest both of the Prince and Princess has been of the most practical and valuable kind. The Nurses' Pension Fund is the first of what may prove to be a series of similar institutions. Statesmen of the highest rank and financiers and philanthropists of eminence have set their minds upon the disestablishment of the poor-house, and the provision of modest comforts with personal liberty for all the well conducted among the working classes in old age. The Royal National Pension Fund, with the Princess of Wales at its head, and the Prince giving it his steady countenance and active support, is an interesting object lesson for Mr. Joseph Chamberlain, Canon Blackley, and those other able and well-intentioned persons who have set their hands to the statesman-like work of teaching and helping the industrial classes to make provision for their own comfort and freedom when they can work no more. It is impossible at present to foresee what far-reaching and beneficent results may spring from the establishment and successful working of this Nurses' Pension Fund. What is clearly demonstrated already is this, that a Donation Bonus Fund of £50,000, which promises to try to add a third or a fourth to the products of the nurses' own savings, has been sufficient to induce a seventh part of the whole nursing profession to commence making serious provision for old age, and that within the short space of three years. If anything like the same results could be depended upon among other classes, we do not hesitate to say that a State pension fund for all orders of the industrial population would work a peaceful revolution of the most advantageous and enduring kind.

Lord Rothschild, in his reply to the Prince of Wales, expressed some surprise that hospital managers had not taken up the Fund with more enthusiasm on behalf of their own nurses. The truth, however, is that a good many hospitals have become federated with the Fund, and a good many more are now arranging bases of federation. Lord Rothschild should remember that all hospital managers are not, like himself, trained in high finance. He saw at once, when the scheme was first put before him, the great value of the Fund to the nurses, and, with him, to see was to act, and to take steps for bringing every nurse in the kingdom under the Fund's beneficent operations. Hospital managers are, as a rule, comparatively inexperienced in general finance. But there is no doubt that, though some of them have not seen the merits of the Pension Fund so promptly and clearly as Lord Rothschild saw those merits, they are now realising and understanding them; and it is certain that so soon as their financial education shall be completed they will be eager to claim for all their nurses a share in the benefits of the Pension and Bonus Funds. Hospital managers should, however, understand that Lord Rothschild has thrown down a friendly challenge to them. As one of the responsible heads of the Pension Fund, he says, in effect, that the Council have done a great and valuable work for nurses; that the work has been endorsed not only by the press and the public, but by the highest persons in the land; and that, moreover, the nurses themselves, though they are but women, have clearly seen and rightly valued the advantages offered to them. Hospital managers, who claim the best services which nurses can render, at the very best time of their lives, ought to feel bound to take all possible steps to secure for their own nurses whatever present and future benefits the Pension Fund may have to offer.

All the distinguished favours which the Prince and Princess of Wales have shown to nurses and their Pension Fund may have a disastrous rather than an advantageous effect if those to be benefited should thereby be led to think that as so much is being done for them, they do not need to do anything for themselves. The real point for nurses to bring their minds to is this, that the Pension Fund is their fund; it has been founded for them, and is to be worked in their interests. It is because the Fnnd is the nurses' own institution and providence, that their Royal Highnesses have taken it under their protecting and fostering care. No more foolish and disloyal conduct could the nurses be guilty of than a want of appreciation of the gracious kindness which has been shown to them in the very highest quarters. What the Princess of Wales asks of them is not some advantage for herself, but that they will show a rational care for their own future interests, and that they will permit her to help them to do so. They cannot but love the Princess for this gracious and steady friendship. They will best show their loyalty and gratitude by claiming the Royal National Pension Fund as their own, and by using it, fully, freely, and on all occasions, as their Royal Highnesses wish them to use it. Last Saturday was a great day for nurses; it is to be hoped that the impressions then made upon their minds may long endure, and yield practical fruit.

